# Endogenous Reverse Transcriptase Inhibition Attenuates TLR5-Mediated Inflammation

**DOI:** 10.1128/mbio.03280-22

**Published:** 2023-01-16

**Authors:** Nicholas Dopkins, Bhavya Singh, Stephanie Michael, Morgan M. O’Mara, Jez L. Marston, Tongyi Fei, Matthew L. Bendall, Douglas F. Nixon

**Affiliations:** a Division of Infectious Diseases, Department of Medicine, Weill Cornell Medicine, New York, New York, USA; University of California, Davis

**Keywords:** human endogenous retrovirus (HERV), long interspersed nuclear element (LINE), toll-like receptors (TLRs), innate immunity, flagella

## Abstract

Transposable elements (TEs) are mobile genomic sequences that encompass roughly 50% of the human genome. Class 1 TEs, or “retrotransposons,” mobilize through the production of an RNA intermediate that is then reverse transcribed to form complementary DNA (cDNA) molecules capable of genomic reinsertion. While TEs are traditionally silenced to maintain genomic integrity, the recognition of immunostimulatory cues, such as those provided by microorganisms, drastically alters host transcription to induce the differential expression of TEs. Emerging evidence demonstrates that the inducible production of TE cDNA is not an inert phenomenon but instead has been coopted by host immunity to facilitate cross talk between host and constituents of the microbiota by agonizing intrinsic antiviral receptors. Here, we demonstrate that immunostimulation of toll-like receptor 4 (TLR4) with lipopolysaccharide (LPS) and TLR5 with bacterial flagella (FLA) alters the expression of retrotransposons, such as human endogenous retroviruses (HERVs) and long interspersed nuclear elements (LINEs). Next, we demonstrate that reverse transcriptase inhibitor (RTi) delivery ameliorates the acute production of the proinflammatory cytokine “tumor necrosis factor alpha” (TNF-α) in response to FLA in a monocytic cell line (THP-1). Collectively, our findings demonstrate that TLR5-mediated cross talk between the host and microbiota is partially dependent on the reverse transcription (RT) of retrotransposons.

## OBSERVATION

Transposable elements (TEs) are highly abundant in the human genome, encompassing roughly 50% of our genetic material (1). TEs are mobile genetic elements that can transpose throughout the genome, and therefore are traditionally repressed to maintain genomic integrity (2). Despite early misconceptions that TEs are purely inert genetic material, recent developments have demonstrated their roles in regulating global transcription (3), mammalian reproduction (4), cellular differentiation (5), the propagation of immunological responses (6, 7), and the pathophysiology of cancers (8) and autoimmunity (9). Our current understanding therefore reevaluates the previous misconception about TEs being “junk” DNA by demonstrating that their cis-regulatory functions and expression are critical to human health and disease.

### The microbiota in health and disease.

The microbiota is a reservoir of communicable microorganisms that reside along mucosal barriers and the skin (10). The microbiota provides critical functions to host health by aiding in metabolism, outcompeting pathogens, and by providing a reservoir of immunomodulatory signals that fundamentally shape immunity (10). Despite the overwhelming abundance of literature demonstrating significant associations between host health and microbial composition, many of the mechanisms that connect microbial abundance to host health remain poorly defined. Emerging evidence suggests that microbial cues dictate the expression and activity of TEs (7, 11), therefore posing novel mechanisms by which the poorly understood “dark genome” mediates host-microbe interactions (12). To better understand how microbiota composition can influence host immunity, we investigated how flagella (FLA), an antigenic motor protein typically possessed by pathogenic microorganisms overabundant during dysbiotic conditions (13), influenced the expression of TEs in human leukocytes through activation of toll-like receptor 5 (TLR5). TLR5 functions as an innate immune receptor of FLA, aids in the maintenance of mucosal homeostasis, and protects the host from metabolic syndromes and dysbiosis-driven inflammation (13, 14).

### TLR stimulation modulates the expression of clas 1 TES.

**FIG 1:**
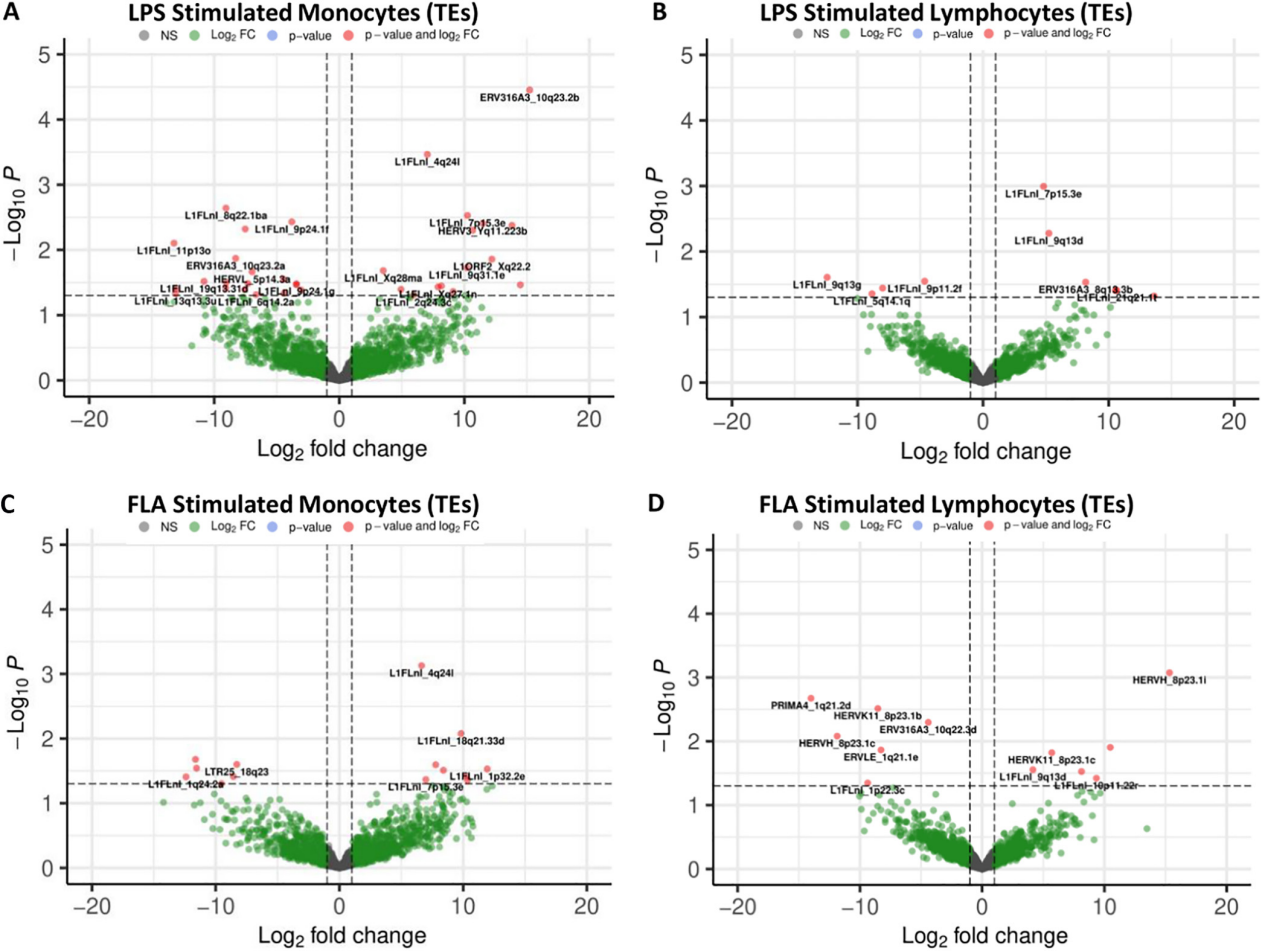
Acute TLR4 and TLR5 activation of PBMCs leads to discrete changes in TE expression. (A) Acute TLR4 activation leads to significant changes in the differential expression of 34 TE loci in the monocytic fraction of healthy donor PBMCs. (B) Acute TLR4 activation leads to significant changes in the expression of 10 TE loci in the lymphocytic fraction of healthy donor PBMCs. (C) Acute TLR5 activation leads to significant changes in the differential expression of 14 TE loci in the monocytic fraction of healthy donor PBMCs. (D) Acute TLR5 activation leads to significant changes in the expression of 12 TE loci in the lymphocytic fraction of healthy donor PBMCs. All TEs were identified as being significantly modulated by possessing a fold change of ≥1 and a *P* value <0.05 determined by the default Wald test parameters of DESEQ2 *(n = *3). A full table of all significantly modified genes and TEs can be found in the supplemental material.

To better define the mechanisms underlying TLR-mediated immunity, we stimulated peripheral blood mononuclear cells (PBMCs) from 3 healthy donors at 2 × 10^6^ live cells/mL with 100 ng/mL lipopolysaccharide (LPS) (Ultrapure LPS from E. coli 0111:B4, InvivoGen) or FLA (Ultrapure flagellin from *S.* Typhimurium, InvivoGen) for 2 h to activate TLR4 or TLR5, respectively. Following activation, we then performed bulk RNA-sequencing on cDNA libraries created from the monocytic and lymphocytic enriched leukocyte fractions (EasySep human monocyte isolation kit, STEMCELL Technologies). Briefly, cDNA libraries were prepared (Illumina stranded total RNA prep with Ribo-Zero Plus, Illumina) from 4.7 ng (monocytic fractions) or 10 ng (lymphocytic fractions) of purified RNA (RNAClean XP, Beckman Coulter). Pair-ended sequencing was performed on an Illumina NovaSeq 6000 using an SP flow cell (2 × 100 bp) with an average output of 34.5 million reads per sample. To determine the effects of TLR activation on TE expression, we quantified TE transcript abundance with locus-specific definition using the bioinformatic pipeline Telescope (15). Briefly, we found that the lymphocytic and monocytic enriched fractions of TLR4- (Fig. 1A and B) and TLR5- (Fig. 1C and D) activated PBMCs display discrete changes in the differential expression of multiple long interspersed nuclear elements (LINE) and human endogenous retrovirus (HERV) elements, suggesting a broad putative role for TEs downstream of TLR signaling. In addition to the changes in TE expression, LPS- and FLA-stimulated PBMC fractions displayed transcriptional changes that signify innate immune activation compared to donor-matched vehicle controls (see Fig. S1 to 4 in the supplemental material). Bar charts demonstrating deviance in the differential expression of TEs (Fig. S5) and a full list of genes/TEs significantly modified (Tables S1 and 2) in the lymphocytic and monocytic fractions of healthy donor PBMCs following acute activation of TLR4 and TLR5 can be found in the supplemental material.

10.1128/mbio.03280-22.1 FIG S1 Acute TLR4 activation leads to significant changes in the differential expression of 257 genes in the monocytic fraction of healthy donor PBMCs. All genes were identified as being significantly modulated by possessing a fold change of ≥1 and a *P* value <0.05 by the default Wald test parameters of DESEQ2 (*n* = 3). Download FIG S1, PDF file, 0.2 MB. Copyright © 2023 Dopkins et al. 2023 Dopkins et al. https://creativecommons.org/licenses/by/4.0/ This content is distributed under the terms of the Creative Commons Attribution 4.0 International license.

10.1128/mbio.03280-22.2 FIG S2 Acute TLR4 activation leads to significant changes in the differential expression of 38 genes in the lymphocytic fraction of healthy donor PBMCs. All genes were identified as being significantly modulated by possessing a fold change of ≥1 and a *P* value <0.05 by the default Wald test parameters of DESEQ2 (*n* = 3). Download FIG S2, PDF file, 0.2 MB. Copyright © 2023 Dopkins et al. 2023 Dopkins et al. https://creativecommons.org/licenses/by/4.0/ This content is distributed under the terms of the Creative Commons Attribution 4.0 International license.

10.1128/mbio.03280-22.3 FIG S3 Acute TLR5 activation leads to significant changes in the differential expression of 119 genes in the monocytic fraction of healthy donor PBMCs. All genes were identified as being significantly modulated by possessing a fold change of ≥1 and a *P* value <0.05 by the default Wald test parameters of DESEQ2 *(n = *3). Download FIG S3, PDF file, 0.1 MB. Copyright © 2023 Dopkins et al. 2023 Dopkins et al. https://creativecommons.org/licenses/by/4.0/ This content is distributed under the terms of the Creative Commons Attribution 4.0 International license.

10.1128/mbio.03280-22.4 FIG S4 Acute TLR5 activation leads to significant changes in the differential expression of 25 genes in the lymphocytic fraction of healthy donor PBMCs. All genes were identified as being significantly modulated by possessing a fold change of ≥1 and a *P* value <0.05 by the default Wald test parameters of DESEQ2 (*n* = 3). Download FIG S4, PDF file, 0.2 MB. Copyright © 2023 Dopkins et al. 2023 Dopkins et al. https://creativecommons.org/licenses/by/4.0/ This content is distributed under the terms of the Creative Commons Attribution 4.0 International license.

10.1128/mbio.03280-22.5 FIG S5 TEs demonstrate significant differential expression following acute TLR4 and TLR5 activation. (A) Bar charts demonstrating the significantly modified expression of individual HERV element loci within the monocytic fraction of TLR4 stimulated PBMCs. (B) Bar charts demonstrating the significantly modified expression of individual LINE element loci within the monocytic fraction of TLR4 stimulated PBMCs. (C) Bar charts demonstrating the significantly modified expression of individual HERV element loci within the lymphocytic fraction of TLR4 stimulated PBMCs. (D) Bar charts demonstrating the significantly modified expression of individual LINE element loci within the lymphocytic fraction of TLR4 stimulated PBMCs. (E) Bar charts demonstrating the significantly modified expression of individual HERV element loci within the monocytic fraction of TLR5 stimulated PBMCs. (F) Bar charts demonstrating the significantly modified expression of individual LINE element loci within the monocytic fraction of TLR5 stimulated PBMCs. (G) Bar charts demonstrating the significantly modified expression of individual HERV element loci within the lymphocytic fraction of TLR5 stimulated PBMCs. (H) Bar charts demonstrating the significantly modified expression of individual LINE element loci within the lymphocytic fraction of TLR5 stimulated PBMCs. Download FIG S5, PDF file, 0.1 MB. Copyright © 2023 Dopkins et al. 2023 Dopkins et al. https://creativecommons.org/licenses/by/4.0/ This content is distributed under the terms of the Creative Commons Attribution 4.0 International license.

10.1128/mbio.03280-22.6 TABLE S1 Alterations in gene expression in the lymphocytic and monocytic fractions of human PBMCs following acute TLR4 and TLR5 stimulation. Download Table S1, XLSX file, 0.03 MB. Copyright © 2023 Dopkins et al. 2023 Dopkins et al. https://creativecommons.org/licenses/by/4.0/ This content is distributed under the terms of the Creative Commons Attribution 4.0 International license.

10.1128/mbio.03280-22.7 TABLE S2 Alterations in TE expression in the lymphocytic and monocytic fractions of human PBMCs following acute TLR4 and TLR5 stimulation. Download Table S2, XLSX file, 0.01 MB. Copyright © 2023 Dopkins et al. 2023 Dopkins et al. https://creativecommons.org/licenses/by/4.0/ This content is distributed under the terms of the Creative Commons Attribution 4.0 International license.

### RTI delivery suppresses TLR5-mediated TNF-α production.

**FIG 2:**
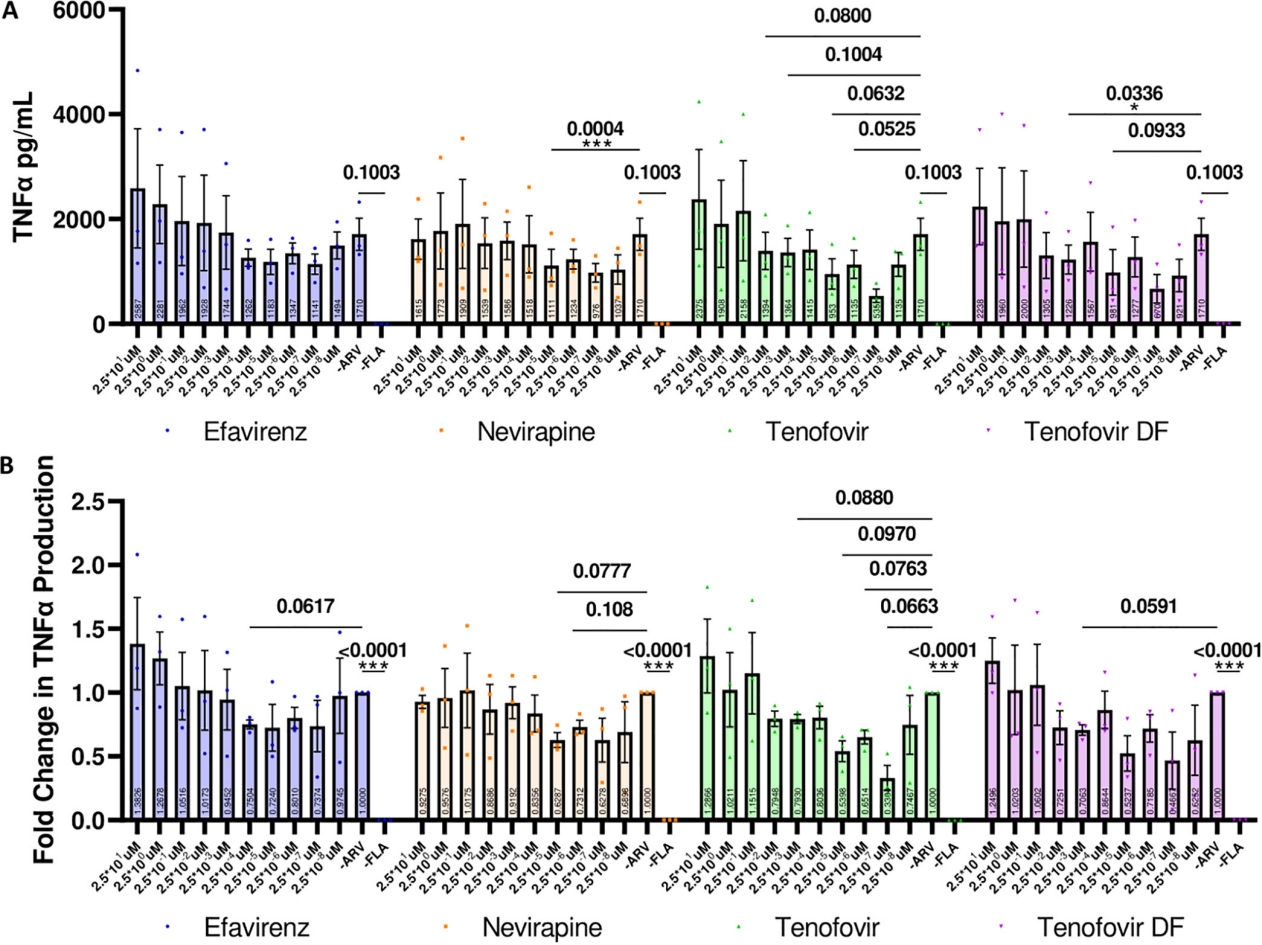
Low-dosage RTi inhibits TLR5-mediated TNF-α production in THP-1 cells. (A) At low doses (2.5 × 10^5^ to 2.5 × 10^8^μM) efavirenz, nevirapine, tenofovir, and tenofovir disoproxil fumarate ameliorate acute TNF-α production in response to TLR5 activation in a dose-dependent manner. Individual values are represented of the average value per experiment, while each experiment was performed with 2 to 3 technical replicates (*n* = 3, Two-way ANOVA with Dunnett's multiple-comparison test with Geisser-Greenhouse's correction for comparing individualized means per condition to the no-antiretroviral [ARV] positive control; F [1.395, 11.16] = 8.818, *P* = 0.0083). (B) Fold change in TNF-α production normalized per experiment (*n* = 3, Two-way ANOVA with Dunnett's multiple-comparison test with Geisser-Greenhouse's correction for comparing individualized means per condition to the no-ARV positive control; F [1.667, 13.34] = 12.23, *P* = 0.0014). All *P* values within the range of 0.1 or lower are displayed and significant *P* values are denoted by a standard scale of *, *P* < 0.05; **, *P* < 0.01; and ***, *P* < 0.001. Dots represent experimental averages with the mean per condition represented at the graph base.

To determine the role of retrotransposon activity in monocytic cells activated by TLR5 stimulation, we next utilized an *in vitro* model of TLR5 stimulation using the monocytic cell line THP-1. THP-1 cells were utilized for these experiments due to their highly sensitive FLA response (16), allowing for an *in vitro* model of acute TLR5 activation. Briefly, we found that acute TLR5 activation of THP-1 cells resulted in increased production of the proinflammatory cytokine tumor necrosis factor alpha (TNF-α) as detected by ELISA (ELISA MAX deluxe set human TNF-α, Biolegend). To determine the role of endogenous reverse transcription (RT) on TNF-α production, we pretreated THP-1 cells with various reverse transcriptase inhibitors (RTis) for 1 h prior to FLA exposure with serial dilutions of both nucleoside (tenofovir, tenofovir disoproxil fumarate [TDF]) and nonnucleoside (efavirenz, nevirapine) RTis. RTis were initially developed for use against HIV-1 infection but have been shown to inhibit the activity of HERV (7, 17, 18) and LINE (19) reverse transcriptase enzymes, therefore providing a tool to determine the role of endogenous cDNA production in the absence of exogenous retroviral infections. RTi preexposure resulted in the amelioration of acute TNF-α production in FLA-stimulated THP-1 cells in a dose-dependent manner (Fig. 2A and B). RTi delivery to THP-1 cells at high doses resulted in a loss of cytokine inhibition, suggesting that this RTi inhibits TNF-α production via a U-shaped hormetic response commonly observed in pharmacological interventions (20). Interestingly, delivery of nucleoside RTis, such as tenofovir and TDF, at low dosages inhibited TNF-α production greater than the delivery of nonnucleoside RTis, such as efavirenz and nevirapine, at equal dosages. This suggests that premature termination of cDNA generation is more efficacious at inhibiting HERV and LINE reverse transcriptase activity than inhibition via allosteric binding with currently available inhibitors in THP-1 cells. Additionally, there was no effect on the “Jurkat” T-cell line, which did not produce detectable levels of TNF-α in response to acute FLA stimulation (data not shown).

### Discussion.

TLRs are innate immune receptors that maintain homeostatic conditions by raising danger signals in response to infection or damage. To better elucidate the role of TEs in innate immunity, we analyzed the capacity for TEs to mediate proinflammatory cytokine production in response to TLR5 activation by FLA, an antigenic bacterial motor protein typically possessed by opportunistic and overt pathogens. TLR5 signaling maintains homeostasis at mucosal surfaces by preventing the onset of microbial dysbiosis, and dysfunctional TLR5 signaling contributes to dysbiotic outgrowth and associated pathologies (13, 14). Our findings demonstrate that the acute activation of TLR4 and TLR5 on primary human leukocytes drives discrete changes in the differential expression of multiple TEs. The observed changes in LINE and HERV expression are not conserved across PBMC fractions or TLRs, suggesting that the discrete changes in expression are personalized to cell-type and TLR-specific pathways. Furthermore, inhibition of the endogenous RT activity suppresses production of TNF-α in response to FLA in THP-1 cells, suggesting that innate immune signaling pathways have coopted endogenous cDNA production to propagate TLR5 signaling. It is currently unclear if RT activity resulting from FLA stems from intracellular signaling downstream of TLR5 activation, cell-cell interactions that follow TLR5 activation, or an upregulation in response to TLR5-mediated inflammatory cytokine production in the media. Collectively, however, these results suggest that TE expression and TLR signaling are more deeply intercalated than previously thought, and that TE activity is critical to the sensing of pathogenic microorganisms. Our data suggest further experimentation is needed to better define this phenomenon but leads to an intriguing hypothesis that repurposing RTis might provide therapeutic relief to conditions characterized by overt hyperactivation of TLR5 signaling, such as cystic fibrosis (21, 22).

### Code availability.

Source code can be found available at https://github.com/nixonlab/dopkins_jan2022.

### Data availability.

The data sets presented in this study can be found online at https://www.ncbi.nlm.nih.gov/bioproject/ under the BioProject number PRJNA903272. Source code can be found available at https://github.com/nixonlab/dopkins_jan2022.
