# Supplementary figures and images for: Endogenous Reverse Transcriptase Inhibition Attenuates TLR5-Mediated Inflammation
Source: mBio. 2023 Jan 16;14(1):e03280-22. doi: 10.1128/mbio.03280-22 (PMC9973353; doi:10.1128/mbio.03280-22)

## LPS Stimulated Monocytes (Genes)

● NS ●  $\text{Log}_2 \text{FC}$  ● p-value ● p-value and  $\text{log}_2 \text{FC}$

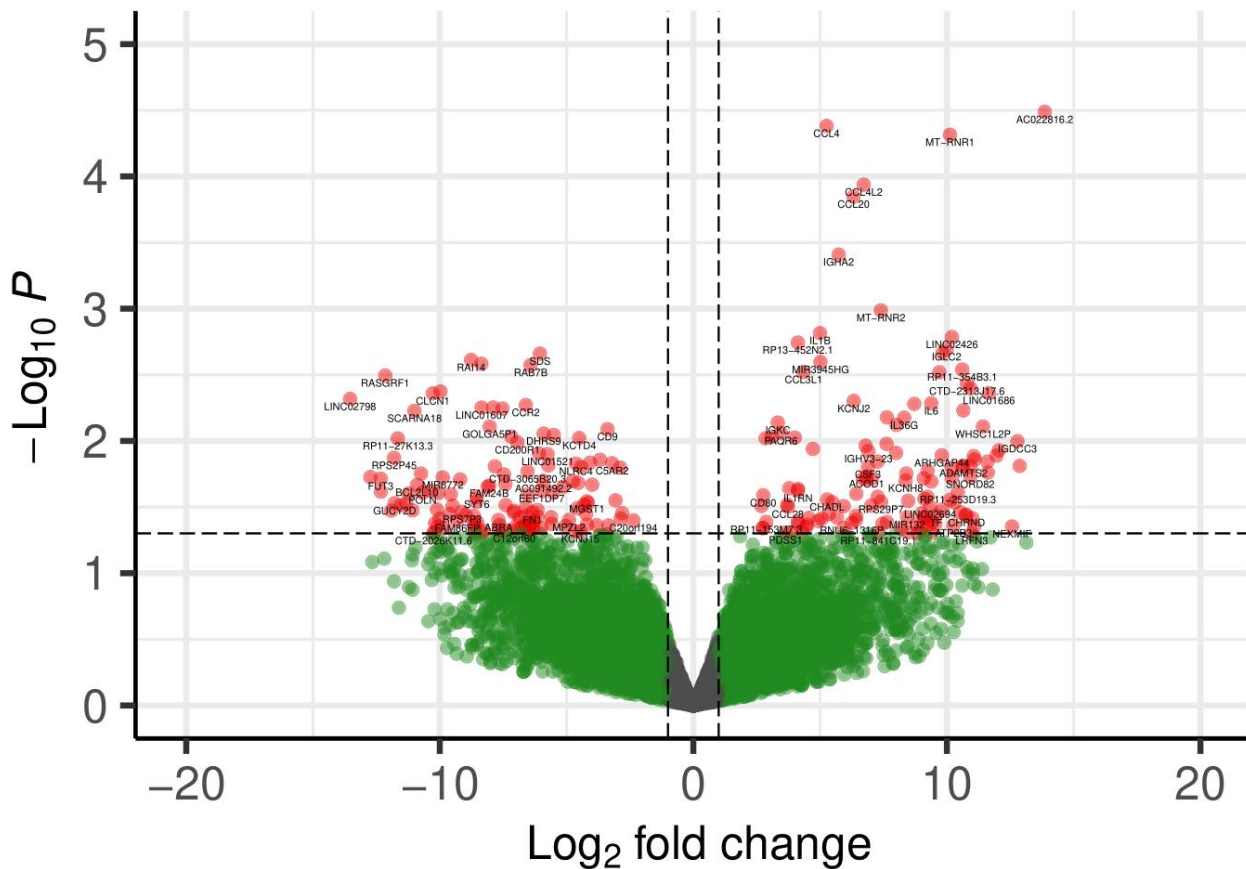

Supplement: FIG S1 [file mbio.03280-22-s0001.pdf]

# LPS Stimulated Lymphocytes (Genes)

● NS ● Log<sub>2</sub> FC ● p-value ● p-value and log<sub>2</sub> FC

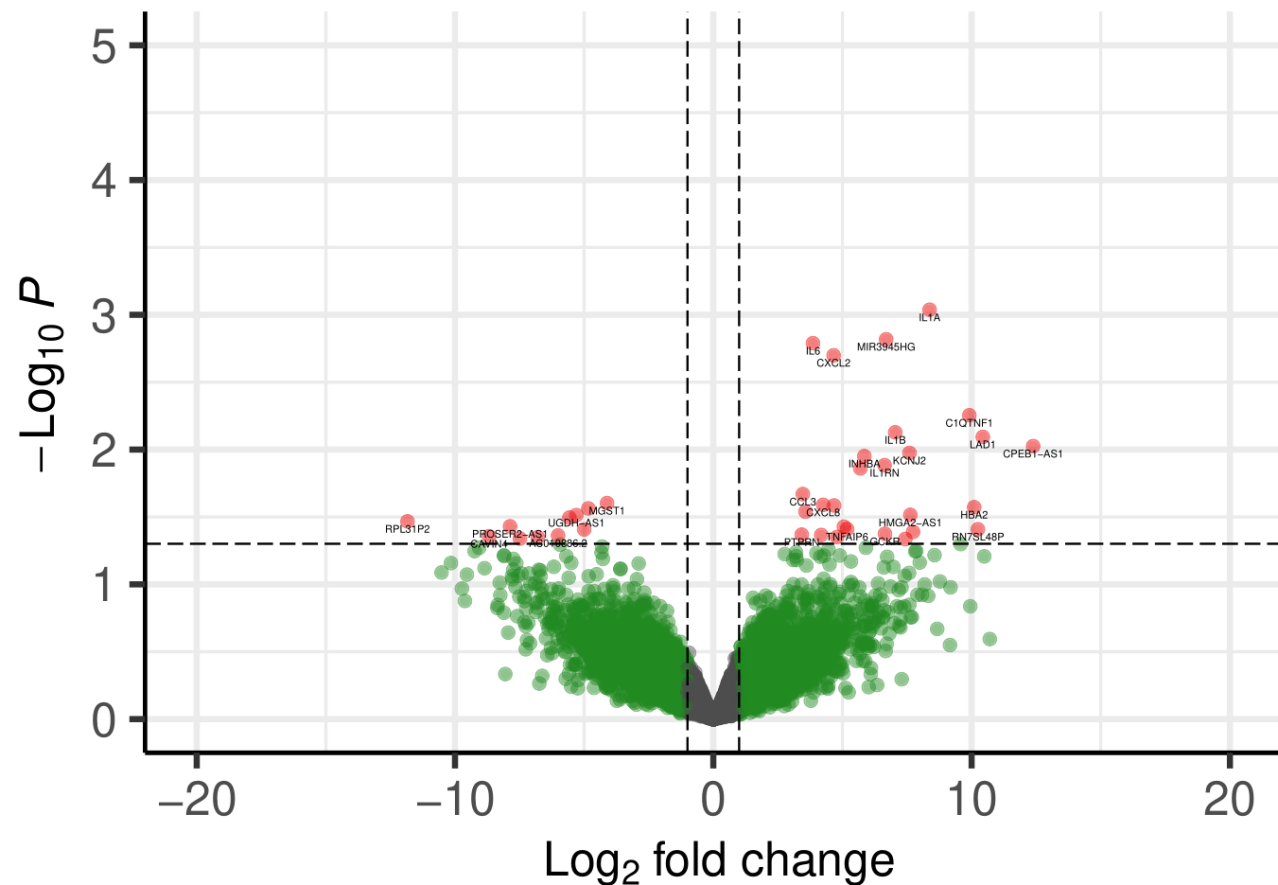

Supplement: FIG S2 [file mbio.03280-22-s0002.pdf]

# FLA Stimulated Monocytes (Genes)

● NS ●  $\text{Log}_2 \text{FC}$  ● p-value ● p-value and  $\text{log}_2 \text{FC}$

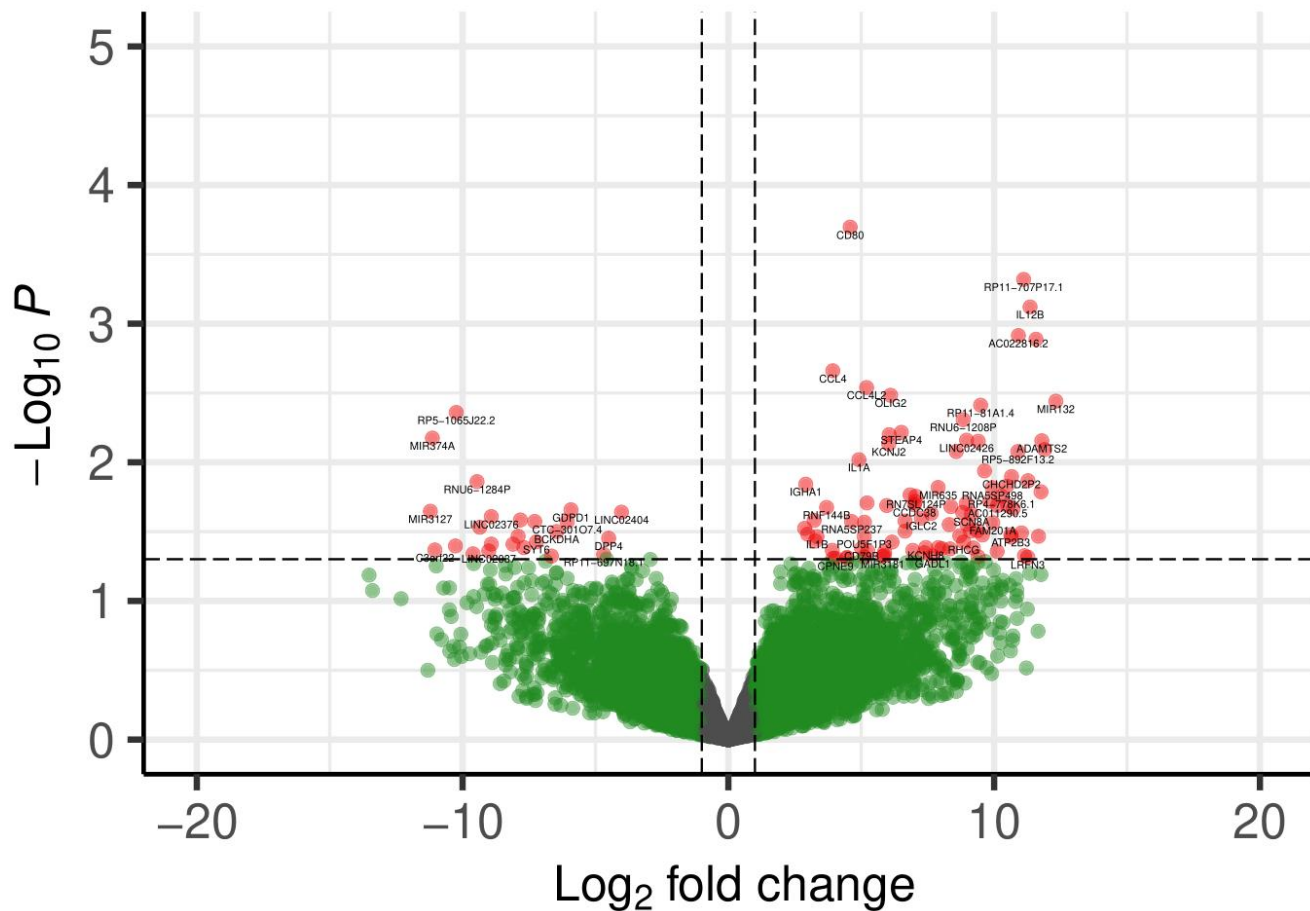

Supplement: FIG S3 [file mbio.03280-22-s0003.pdf]

# FLA Stimulated Lymphocytes (Genes)

● NS ● Log<sub>2</sub> FC ● p-value ● p-value and log<sub>2</sub> FC

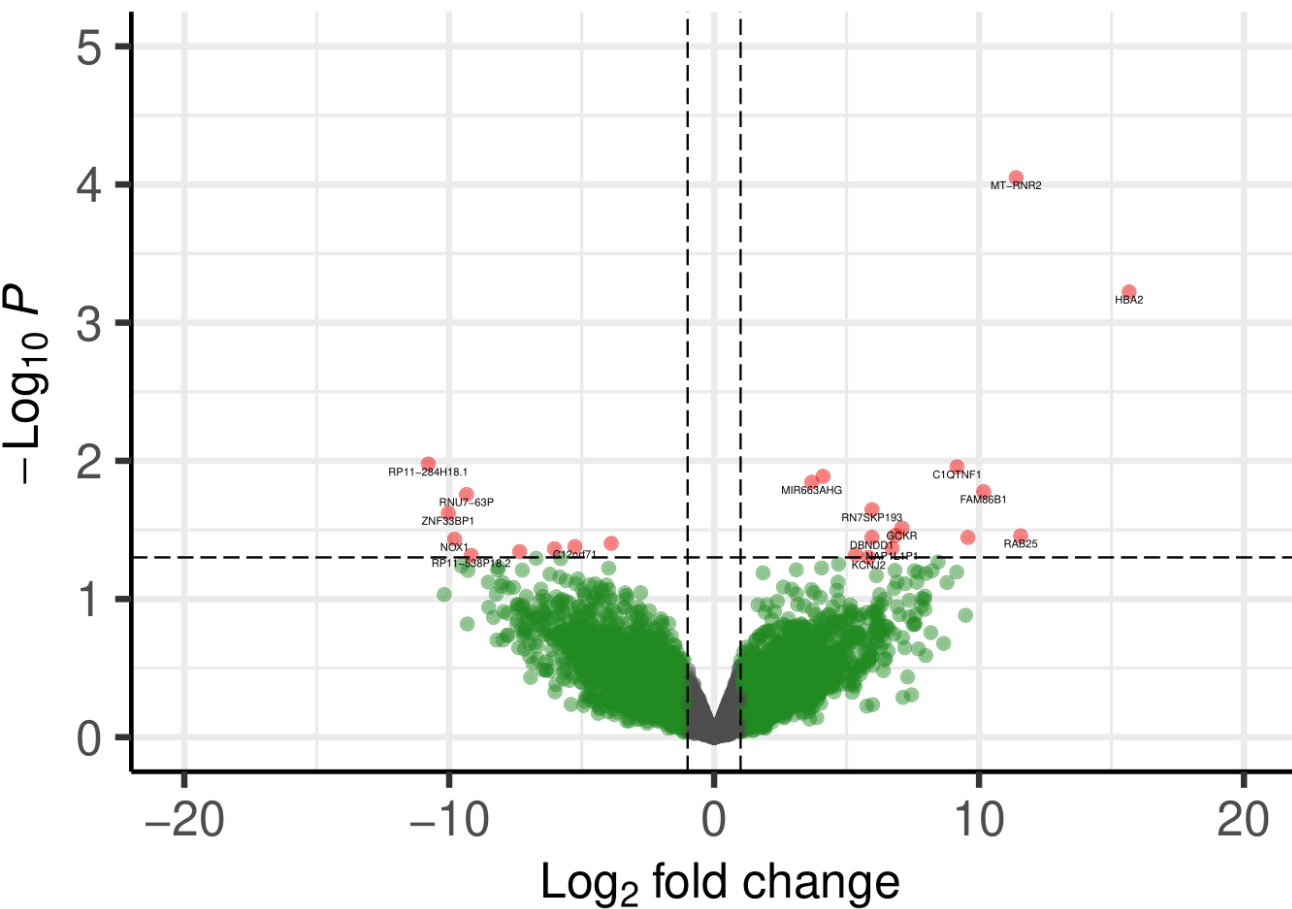

Supplement: FIG S4 [file mbio.03280-22-s0004.pdf]

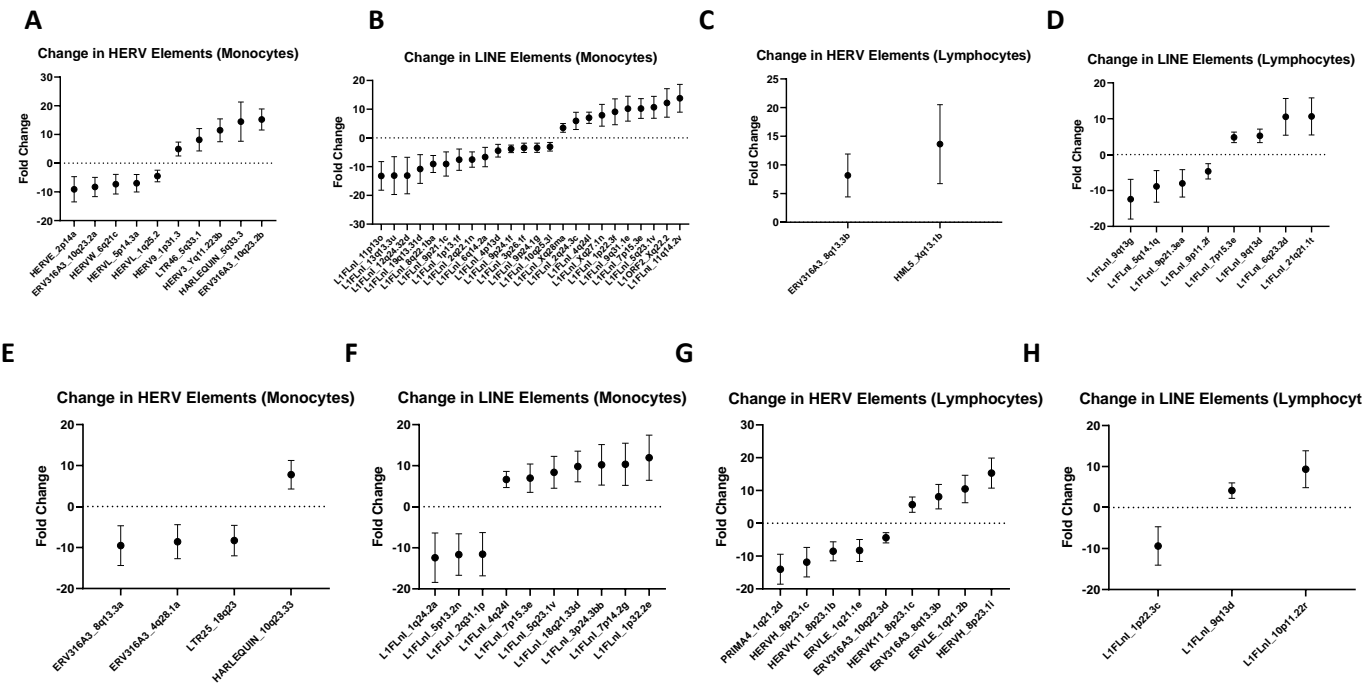

Supplement: FIG S5 [file mbio.03280-22-s0005.pdf]
